# A Lightweight Vision-Based Emotion Sensing Framework for Assistive Healthcare Robotics

**DOI:** 10.3390/s26092865

**Published:** 2026-05-03

**Authors:** Hosam Zolfonoon, Helder Jesus Araújo, Lino Marques

**Affiliations:** Institute of Systems and Robotics, University of Coimbra, 3030-290 Coimbra, Portugal; helder@isr.uc.pt (H.J.A.); lino@isr.uc.pt (L.M.)

**Keywords:** facial expression recognition (FER), facial feature mapping (FFM), telepresence robots in elderly care, facial landmark normalization, machine learning, MediaPipe

## Abstract

Facial expression recognition (FER) for assistive and telepresence robotics remains challenging under resource-constrained conditions because landmark normalization is often unstable, many datasets have limited variability, and full facial landmark sets introduce redundancy. This paper proposes a lightweight, privacy-preserving FER framework for assistive healthcare robotics based on geometric facial landmarks rather than raw RGB images. The objective is to improve recognition robustness and deployment suitability on low-power edge devices through two complementary contributions: a revised nose-centered landmark normalization method and an optimized Facial Feature Mapping, FFM-L03. The proposed normalization replaces the expression-sensitive upper-lip reference with a geometrically stable nose-center anchor, while FFM-L03 combines FACS-guided anatomical priors with ANOVA F-score, LASSO, PCA, and t-SNE/UMAP to retain 60 informative landmarks. In addition, a heterogeneous Freepik dataset was constructed to increase variability in lighting, background, resolution, and subject appearance. Experimental evaluation across 15 landmark groups, four datasets, and four classifiers shows that the proposed method consistently improves performance over prior landmark configurations, achieving gains of up to 22.4 percentage points over the Ciraolo baseline and 22.1 percentage points over the full-landmark baseline in accuracy, precision, recall, and F1-score, while maintaining lightweight operation. These results demonstrate that principled normalization and targeted landmark selection can substantially improve FER for real-time, privacy-aware assistive robotic systems.

## 1. Introduction

The integration of telepresence robots in senior care has shown significant potential in improving the quality of life (QoL) for older adults. These robots not only enable continuous remote health monitoring and social interaction but also play a crucial role in addressing the emotional and cognitive needs of this population. One of the key advancements in this field is the use of FER technology, which allows robots to detect and respond to the emotional states of users in real time. This capability can provide personalized emotional support, helping to reduce loneliness and mental distress among seniors. Furthermore, developments in information and communication technologies (ICT), such as cloud and edge computing, have enhanced the efficiency and scalability of telepresence systems. These advancements ensure low-latency, secure, and real-time data processing, enabling healthcare providers to monitor aging individuals effectively and intervene when necessary.

A hybrid edge-cloud computing framework for telepresence leverages the strengths of both edge and cloud computing to enhance remote communication and monitoring in real-time applications, such as healthcare. In this framework, data processing is distributed between edge devices, which provide low-latency, localized processing close to the data source, and cloud platforms, which handle more intensive computations and long-term data storage. The edge layer ensures fast responses and real-time interactions by processing sensory inputs and video streams locally, while the cloud layer manages larger-scale analysis and coordination. This architecture is especially beneficial for telepresence applications, enabling seamless user experiences with minimal delays and improved reliability [1,2,3,4].

The integration of telepresence robots in senior care has demonstrated considerable potential for improving the quality of life (QoL) of older adults. Beyond physical monitoring, robots are increasingly expected to assist with the emotional and cognitive well-being of elderly individuals, particularly in long-term care scenarios where loneliness and social isolation are common. Consequently, the ability of robotic systems to perceive and interpret human emotional states has become an important component of effective human–robot interaction in assistive healthcare environments.

Building on this technological foundation, telepresence robots extend these capabilities into real-world healthcare applications. FER has therefore emerged as a promising technology for enabling robots to detect emotional cues during communication. FER systems use computer vision and machine learning techniques to analyze facial movements and micro-expressions in order to infer underlying emotional states. When integrated into telepresence platforms, FER can enrich remote interactions by providing contextual information about a user’s emotional condition, which is particularly valuable in domains such as telemedicine, education, and assistive care. For example, monitoring emotional responses can help healthcare professionals assess stress, discomfort, or psychological well-being during remote consultations [5,6].

Despite the growing interest in emotion-aware telepresence systems, the integration of FER into real-world robotic platforms remains limited. Many existing telepresence systems focus primarily on communication and navigation capabilities, while emotional perception is often omitted or treated as a secondary feature. As a result, current systems frequently lack the ability to capture non-verbal emotional cues that are essential for natural human interaction. The absence of emotional awareness can lead to incomplete communication, reduced engagement, and limited responsiveness in remote healthcare scenarios. Addressing this limitation represents a significant opportunity for improving human–robot interaction and emotional intelligence in telepresence environments [7,8].

Another critical limitation arises from the computational requirements of many modern FER methods. State-of-the-art FER approaches frequently rely on deep neural networks trained directly on RGB facial images. Although these models can achieve high recognition accuracy, they typically require high-resolution inputs, GPU-level computation, and large training datasets. Such requirements are incompatible with many robots used in healthcare, which often operate on low-power embedded hardware with limited computational resources. Moreover, RGB-based FER pipelines frequently depend on cloud processing, increasing system latency and raising privacy concerns related to the transmission and storage of identifiable facial images.

To address these limitations, alternative approaches that rely on geometric facial representations have gained increasing attention. Instead of processing full facial images, landmark-based methods represent facial expressions using a set of key facial points that describe the geometric structure of the face. This representation significantly reduces computational complexity while preserving essential information about facial movements. Landmark-based FER pipelines are therefore particularly attractive for resource-constrained platforms, as they enable efficient real-time processing while minimizing data transmission requirements.

In this work, the facial camera of the telepresence robot is treated as a vision-based sensing device that continuously acquires facial signals during interaction. The captured visual data are processed through a landmark-based signal-processing pipeline that extracts facial landmarks and converts them into structured geometric features. These features are then analyzed using lightweight machine learning models to infer emotional states in real time. By transforming raw visual input into anonymized landmark coordinates, the proposed approach also enhances privacy protection by avoiding the storage or transmission of identifiable facial images. From a sensing perspective, the framework can be interpreted as an AI-enabled perception system that integrates image sensing, signal processing, and machine learning to enable emotion recognition in assistive robotics.

Nevertheless, several challenges remain in existing landmark-based FER pipelines. First, many normalization techniques used for aligning facial landmarks are sensitive to facial pose variations and expression changes. For instance, some previous methods rely on landmarks located around the mouth region, such as the upper lip, as reference points for geometric alignment. Because these landmarks move significantly during facial expressions, they may introduce distortions and inconsistencies in the normalized coordinate space. Second, widely used FER datasets often exhibit limited diversity in terms of demographic attributes, lighting conditions, and image quality, which restricts the generalization capability of trained models. Third, many existing studies do not systematically evaluate the informativeness of individual facial landmarks, resulting in feature vectors that may contain redundant or irrelevant information. Finally, evaluations of classifier performance are frequently limited to a small number of models, making it difficult to determine which algorithms are most suitable for resource-constrained applications.

To address these limitations, this work investigates three critical components of landmark-based FER systems: training datasets, machine learning models, and facial feature representations. Three widely used FER datasets are selected for evaluation, and an additional heterogeneous dataset is constructed by combining samples from multiple sources in order to reduce potential dataset bias and increase demographic diversity. For feature representation, facial landmarks extracted using the MediaPipe Face Mesh framework are converted into structured feature vectors referred to as FFM. Different FFM configurations are systematically compared to identify the most informative FFM while maintaining computational efficiency.

### 1.1. Motivation

To enable reliable real-time emotion recognition in assistive and telepresence robotics, this work addresses the fundamental limitations of existing FER pipelines when deployed on resource-constrained platforms. Conventional FER approaches, particularly those relying on deep neural networks, require high-resolution RGB inputs, GPU-level computation, and cloud-based processing, making them unsuitable for low-cost robotic systems commonly used in healthcare environments [9]. These constraints restrict on-device inference, increase latency, and raise privacy concerns due to the transmission of identifiable facial images.

This paper is motivated by the need for a computationally efficient, privacy-preserving, and sensor-centric FER framework that can operate entirely on lightweight edge hardware. By treating the robot’s facial camera as a vision-based sensor and converting raw images into anonymized landmark signals, the proposed system enables FER through a compact geometric representation rather than pixel-level processing. This approach significantly reduces computational load while maintaining discriminative power, making it feasible for embedded robotic platforms [10].

Finally, to ensure compliance with privacy and data-protection requirements in healthcare applications, all processing is performed locally on secure edge devices. No facial images are stored or transmitted, and only non-identifiable landmark coordinates are used for inference. This design aligns with GDPR principles and supports safe deployment in sensitive environments such as elderly care and telemedicine [11,12].

### 1.2. Novelty and Contributions

The novelty of this work lies in the joint design of a geometrically stable normalization strategy and a compact, anatomically guided landmark representation for lightweight FER in assistive robotics. First, the proposed method replaces the expression-sensitive upper-lip reference used in prior landmark-based normalization with a stable nose-centered anchor and axis-independent scaling, producing a more consistent coordinate space across facial expressions and poses. Second, the proposed FFM-L03 integrates FACS-based anatomical knowledge with ANOVA F-score, LASSO, PCA, and t-SNE/UMAP through an intersection-based rule to select 60 informative landmarks, with particular emphasis on the lower-face regions that are highly relevant for emotional deformation. Third, the study validates the framework across multiple benchmark datasets together with a heterogeneous custom dataset and shows that the proposed design improves FER performance by up to 26.1 percentage points over the Ciraolo baseline while preserving privacy and edge-deployment suitability. These contributions position the proposed framework as a lightweight and practically deployable alternative to computationally intensive RGB-based FER approaches.

## 2. Literature Review

FER began with Darwin’s 1872 work on the universality of facial expressions. In the 20th century, Ekman’s FACS and Izard’s emotion theory mapped expressions to muscle movements and basic emotions. Later, advances in computer vision and standardized datasets enabled automated analysis. Recently, CNNs and RNNs have boosted FER accuracy, expanding its use across industries like healthcare, marketing, and security [9,13].

Almeida et al. [14] emphasize FER’s role in telepresence robotics, helping robots interpret users’ nonverbal cues and adapt behavior [14]. This is especially useful in healthcare and therapy, improving empathy and social interaction [14]. Vasylkiv et al. created Haru, a telepresence robot using a MobileNet-based CNN to detect seven emotions [15]. Integrated with a reinforcement learning system, Haru learns to respond better over time, easing the teleoperator’s workload [15]. Swadha et al. used MediaPipe Face Mesh to extract 470 key points, improving emotion detection in online classes through neural networks [16]. Mukhriddin et al. also found MediaPipe effective in recognizing student emotions in real time [17].

Davide et al. integrated Emotional AI with FER for remote therapy, helping clinicians monitor emotional states. They optimized detection pipelines using MediaPipe and various classifiers and datasets [9]. Naseer et al. examined robots like Giraff and RP-Vita during COVID-19. These robots supported remote consultations and hygiene by reducing physical contact, using various control systems [18]. Păvăloiu et al. reviewed THRs in elderly care, highlighting their role in remote monitoring, consultations, and reducing loneliness, the paper also explored system design and functionality [19]. Janika et al. found TPRs reduce staff workload but face technical and user-acceptance challenges [20]. Zhang and Hansen noted accessibility and usability barriers in special-needs robots, calling for future improvements [21]. Smith et al. proposed a scoping review on robots in aged care, focusing on how they alleviate loneliness by maintaining family connections during the pandemic [22]. Ruiz-del-Solar et al. introduced Pudu, a telepresence robot used in Chilean hospitals during COVID-19 [10]. It enabled safe patient-staff interaction and supported emotional care in ICUs [10] (Table 1).

## 3. Methods

This chapter presents the proposed FFM framework, hereafter referred to as FFM-L03, designed to enhance the robustness, discriminability, and computational efficiency of FER in resource-constrained environments. The method introduces two complementary innovations: (i) a revised landmark normalization strategy that improves geometric stability across facial poses and expressions, and (ii) an optimized FFM derived from the intersection of empirical and data-driven selection criteria. The framework is specifically tailored for telepresence and assistive robotic systems, where real-time facial analysis must be performed on lightweight edge-computing hardware.

The proposed system operates on visual data captured by a camera mounted on the robot. This camera functions as the primary vision sensor, continuously acquiring facial images from the user during interaction. The captured frames constitute the raw sensor measurements from which facial landmarks are extracted using the MediaPipe Face Mesh algorithm.

### 3.1. Revised Normalization Method

Traditional normalization techniques for landmark-based FER, such as the one proposed by Ciraolo et al. [9], use the upper lip center as the reference point for alignment. While effective under controlled conditions, this reference point is highly sensitive to mouth opening, speaking, yawning, or smiling, causing unstable coordinate shifts during expression changes. These instabilities propagate through the normalization process and degrade classification performance, as shown later in Section 4. To address this limitation, a revised geometric normalization strategy is introduced based on two modifications:

(a) Stable Reference Point: The center of the nose replaces the upper lip as the spatial anchor. The nose center is minimally affected by expression intensity, anatomically stable across subjects, invariant across most pose variations, and consistently detected by MediaPipe landmarks. Let’s (x_i_, y_i_) denote the coordinates of the i-th landmark and (x_n_, y_n_) the coordinates of the nose center. All landmarks are translated using:

$$x_{i}^{\prime }=x_{i}-x_{n}\;\;\;\;y_{i}^{\prime }=y_{i}-y_{n}$$

(b) Axis-Independent Scaling: To eliminate variations due to image size, face size, or camera distance, the translated coordinates are scaled using the maximum absolute displacement along each axis:

$$x_{i}^{\prime \prime }=\frac{x_{i}^{\prime }}{max\left|x^{\prime }\right|}\;\;\;\;y_{i}^{\prime \prime }=\frac{y_{i}^{\prime }}{max\left|y^{\prime }\right|}$$

These yields normalized landmark coordinates bounded within [−1,1], ensuring invariance to scale, camera distance, and resolution. The resulting coordinate space is far more stable than lip-centered normalization, especially for expressions with strong mouth deformation (happiness, surprise).

### 3.2. Proposed FFM

**Figure 1:**
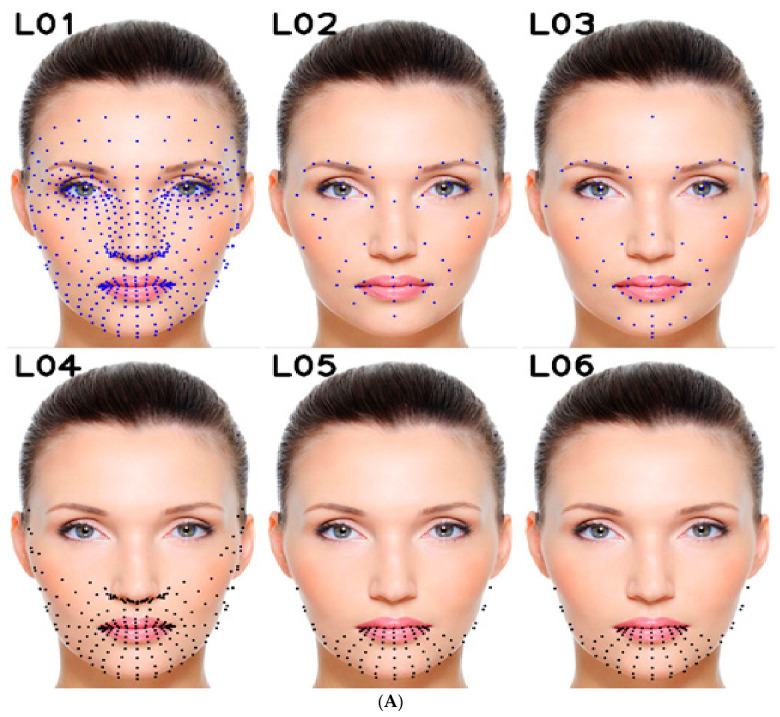

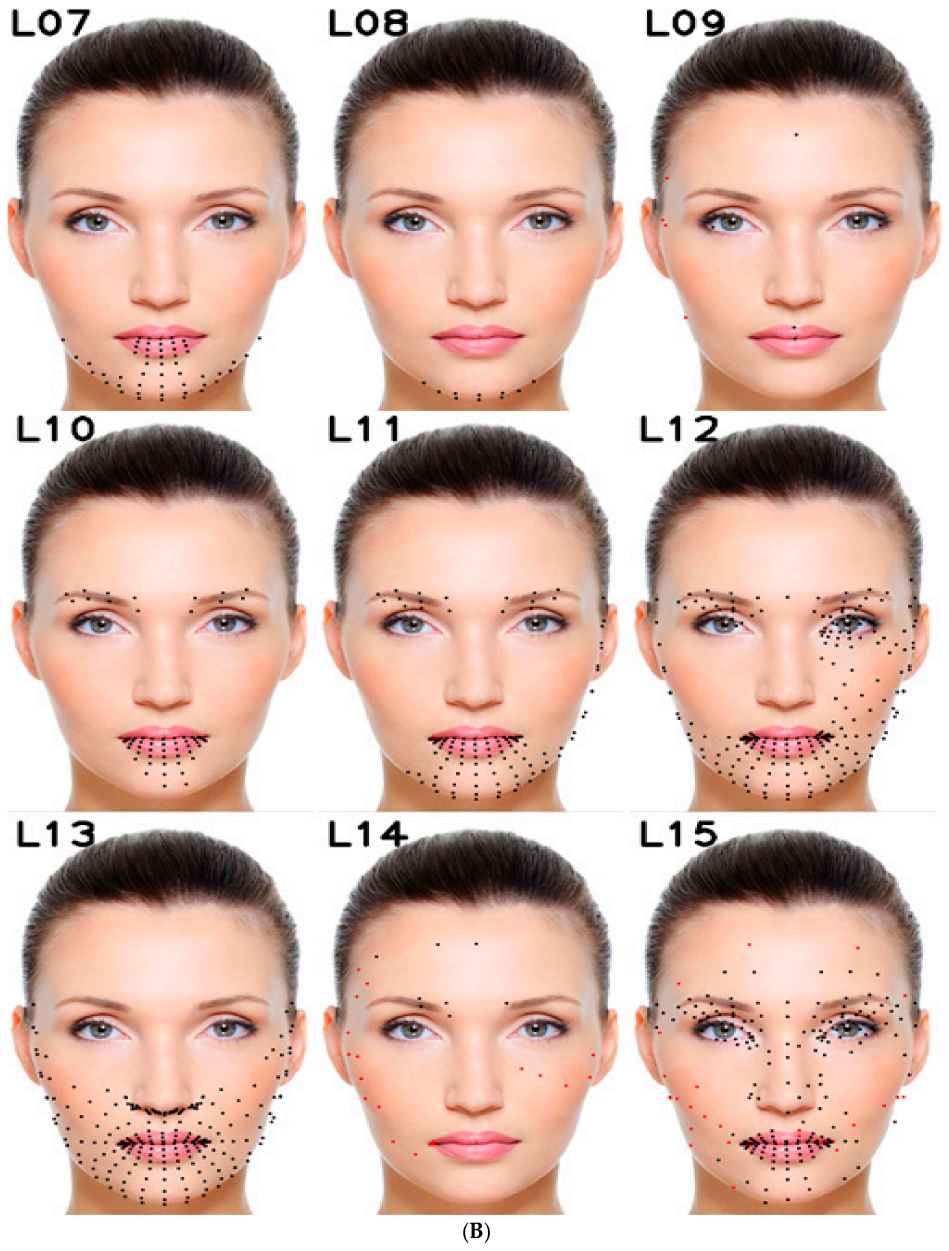
(**A**,**B**). Visualization of features list on sample images (landmarks including X and Y → ●, only X → ●, only Y, → ●).

The visualization in Figure 1A,B further clarifies how different feature-selection strategies emphasize distinct facial regions and, therefore, why FFM-L03, based on the L03 group in Table 2, was designed as a more meaningful compromise. Building on the revised normalization, FFM-L03 aims to improve discriminative power while limiting computational cost. Although MediaPipe Face Mesh provides 478 two-dimensional landmarks, corresponding to 956 coordinate features, using the complete set is computationally inefficient and introduces substantial redundancy [16,17].

For this reason, the proposed configuration was guided by the Facial Action Coding System (FACS), prioritizing regions where emotion-related action units are most strongly expressed. These include the lips and perioral area (e.g., AU12, AU25, AU26), the chin and jawline curvature (e.g., AU17), and the lower-face muscular tension associated with sadness, disgust, or contempt (e.g., AU15) [23,24,25]. This anatomical rationale is also visually supported by Figure 1A,B, where multiple statistical methods consistently retain landmarks around the mouth, lower lip, chin, and adjacent cheek regions, indicating that these areas carry strong emotion-related deformation patterns.

Table 2 provides a spatial and anatomical interpretation of all feature subsets, allowing assessment of whether mathematically selected landmarks correspond to meaningful facial regions involved in emotional expression. The associated selection methods and the number of retained features are summarized subsequently in Table 3.

The feature groups illustrated in Figure 1A,B and summarized in Table 3 provide a structured overview of how each selection method behaves. The variance-threshold groups L04–L08 progressively reduce the feature space from 241 to 29 landmarks as the threshold increases, concentrating mainly on the mouth, lower lip, chin, and lower facial contour. This behavior aligns with the goal of preserving highly variable regions while discarding stable ones, improving efficiency and robustness in FER applications [26]. However, Figure 1A,B also shows that variance-based selection suppresses landmarks in relatively stable but still expressive regions, such as parts of the peri-ocular and eyebrow areas, indicating that high variance alone is not sufficient to guarantee discriminative relevance.

A similar limitation is observed for the correlation-matrix-based selection. The L09 group, obtained by removing highly correlated features using a threshold of 0.8, retains only 10 landmarks [27]. These points are sparse and scattered, lacking concentration in coherent expressive facial regions. This confirms that redundancy reduction alone may remove spatial continuity necessary for representing meaningful facial deformation patterns.

In contrast, the ANOVA F-score groups L10–L12 exhibit a much more expression-oriented spatial distribution. Since ANOVA ranks features based on between-class versus within-class variance [28], the selected landmarks are concentrated in the mouth, eyes, and eyebrows, with increasing regional coverage as the number of retained features grows. These regions closely correspond to FACS-defined action units, confirming that ANOVA captures features with strong class-discriminative value.

The behavior of LASSO in L13 further supports this observation. By enforcing sparsity, LASSO preserves predictive structure while eliminating redundant features [29]. The selected landmarks concentrate around the mouth, chin, lower cheeks, and nasolabial region, forming a structured and anatomically meaningful subset.

Dimensionality-reduction methods show different characteristics. PCA (L14) preserves global variance, but results in a very small number of representative features concentrated near the central facial region, leading to limited interpretability for emotion-related deformation [30]. In contrast, t-SNE and UMAP (L15) preserve nonlinear relationships and produce a dense distribution of landmarks across expressive facial regions [31]. However, many selected features correspond to only one coordinate dimension (x or y), limiting their direct applicability for structural facial representation.

Taken together, these observations justify the construction of FFM-L03. Rather than relying on a single selection criterion, FFM-L03 combines FACS-guided anatomical priors with multiple complementary analytical methods, including ANOVA for discriminative power, LASSO for sparsity, PCA for variance preservation, and t-SNE/UMAP for nonlinear structure. This integrated approach ensures that selected landmarks are both anatomically meaningful and statistically informative.

To ensure reproducibility, the construction of FFM-L03 is formalized as a stepwise intersection-based selection procedure. First, an empirical candidate pool is defined based on FACS-inspired anatomical regions, including the eyebrows, peri-ocular area, cheeks, mouth, lower lip, chin, and lower facial contour. Second, independent feature subsets are generated using ANOVA, LASSO, PCA, and t-SNE/UMAP, all computed exclusively on the training data. Since these methods operate at the coordinate level, their outputs are converted to landmark-level selections by grouping x and y coordinates.

For each landmark, the number of analytical methods selected for it is counted. The final subset retains only landmarks that satisfy two conditions simultaneously: (i) inclusion in the empirical (FACS-guided) candidate pool, and (ii) selection by at least two analytical methods. If more than 60 landmarks satisfy these conditions, they are ranked based on frequency of selection and anatomical relevance, and the top 60 are retained.

The resulting FFM-L03 subset contains 60 landmarks, maintaining dimensional comparability with Ciraolo’s FFM (L02) while providing a more balanced and expressive spatial distribution. In particular, it increases landmark density in the chin and lower-face region, which Figure 1A,B consistently identifies as highly informative across datasets and methods. The selected landmarks cover key expressive regions, including the eyebrows, eyes, cheeks, mouth, and chin, ensuring both anatomical relevance and discriminative capability. The full list of selected landmark indices is provided in Table 4.

To ensure a fair and unbiased evaluation, the dataset is first split into training and test subsets. All feature selection procedures are performed exclusively on the training data. Model performance is then evaluated using cross-validation within the training set, while the held-out test set is used only for final validation. The selected feature subsets remain fixed when applied to validation and test data, preventing information leakage and ensuring that the reported results reflect true generalization capability across different feature configurations (L01, L02, and L03).

### 3.3. Differences in Metric Definition and Interpretation

To quantify the relative performance gain introduced FFM-L03, a defines difference metric that measures the improvement of FFM-L03 over baseline landmark configurations. Let $L_{s,c,m}$ denote the performance score obtained using landmark set s, classifier c, and evaluation metric m.

The difference is computed as

$${Difference}_{s,c,m}={L03}_{c,m}-L_{s,c,m}\;\;\;\;\;s\in \left\{L01,L02\right\}$$

where L01 corresponds to the full landmark set, L02 corresponds to the Ciraolo landmark configuration.

L03 corresponds to the proposed empirical landmark selection, c= {SVM, DT, RF, MLP} denotes the classifier, m= {Accuracy, Precision, Recall, F1−score} denotes the evaluation metric. A positive difference value indicates FFM-L03 outperforms the corresponding baseline configuration for the same classifier and metric.

### 3.4. Datasets

In machine learning research, the dataset serves as a foundational component, often collected and preprocessed in earlier studies and subsequently used in various investigations. Acquiring data, particularly facial images annotated with emotional labels, is a labor-intensive process. To ensure model robustness in this paper, a custom dataset was curated by collecting images from an online repository using a single expert annotator (the corresponding author). (The dataset is publicly available at EmotionNet-6 Realistic Facial Emotion Dataset: https://www.kaggle.com/datasets/isrcoimbra/emotionnet-6-realistic-facial-emotion-dataset (accessed on 17 April 2026), where licensing and usage conditions are specified. Images were sourced from Freepik.com under its royalty-free licensing terms, including periods of paid subscription to ensure compliant access, and explicit confirmation was obtained from the provider for research use. Only non-AI-generated images were included, and the dataset documentation has been updated to clearly acknowledge Freepik.com as the source.) Facial image datasets generally fall into two categories: (i) frames extracted from videos and (ii) standalone images. In both cases, images are typically labeled by either experiment participants or trained psychologists. To evaluate and compare the results, the FFM developed in this research was applied to widely used facial image datasets, including FER-2013, KDEF, and their various combinations, which consist exclusively of static images. The distribution of images per emotional category for these datasets is provided in Table 5.

The following provides a brief overview of the four main datasets referenced in this paper:

#### 3.4.1. Freepik Dataset

Emotion labels define the target categories for FER, spanning core emotions (anger, fear, happiness, sadness, and surprise) [24] and, especially in HRI and industrial contexts, neutral states, finer-grained categories such as contempt, and intensity levels from low to high that help structure datasets and improve model learning [25]. In practice, these labels map to characteristic facial cues that guide annotation and model decisions, including happiness (smile, raised cheeks), sadness (frown, drooping eyelids, downturned mouth), anger (furrowed brows, tightened jaw, flared nostrils), surprise (widened eyes, raised eyebrows, open mouth), fear (wide eyes with drawn-together brows, tense muscles), disgust (wrinkled nose, raised upper lip, lowered chin), contempt (asymmetric mouth raise, slight eye roll), confusion (furrowed brow, puzzled look), interest (raised eyebrows, dilated pupils), and amusement (smile with laugh lines), which are critical for interpreting social signals and for reliable FER training and evaluation [26]. The Freepik dataset was developed for this research to achieve a balanced representation of images across emotional states; images were sourced from Freepik.com without constraints on resolution, facial depth, lighting, background, age, gender, ethnicity, or facial diversity of subjects to enhance robustness and generalizability, and classes were annotated using the cues mentioned above by one of the authors—for example, marking an image as “Happy” when smiling, twinkling eyes, and raised cheeks were present; to ensure balance, 100 images were gathered per emotion across six categories and then mirrored for data augmentation, yielding 200 images per class and a final dataset of 1200 images.

**Figure 2:**
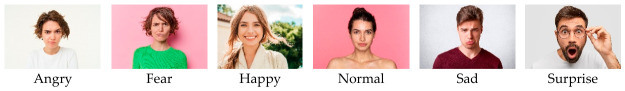
Example images from each class in the Freepick dataset.

A limitation of this dataset is that, although care was taken to exclude explicitly AI-generated images, the use of stock photography may still introduce bias due to staged expressions, controlled conditions, or post-processing enhancements (e.g., lighting, retouching), which could affect generalization to spontaneous, real-world facial expressions encountered in robotic interaction scenarios (Figure 2).

#### 3.4.2. FER-2013

The Facial Expression Recognition 2013 (FER-2013) dataset was created as part of a Kaggle competition on challenges in representation learning, introduced by Goodfellow et al. [31]. The FER 2013 dataset comprises 35,887 grayscale, 48 × 48 pixel images of faces categorized into six emotions: anger, fear, sadness, surprise, and neutral. Initially, the dataset included seven categories, but the “disgust” class, with only 547 samples, was omitted due to its extremely low representation, which contributed to significant imbalance. However, even after removing the “disgust” class, the dataset remains imbalanced, with certain emotions being overrepresented. To address this issue, an undersampling process has been applied to reduce the dominance of overrepresented classes, ensuring more balanced learning across emotions. This strategy improved the model’s ability to generalize an enhanced fairness in recognizing diverse facial expressions [31].

**Figure 3:**
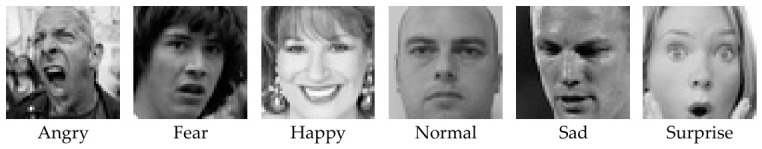
Example images from each class in the FER 2013 dataset.

Undersampling is a data balancing technique that addresses class imbalance by reducing samples from the majority class. In this work, it is implemented using scikit-learn, where a random subset of the majority class is selected to match the minority class size. This helps mitigate model bias toward dominant classes and improves classification performance on imbalanced data. However, undersampling may discard useful information, potentially affecting the model’s ability to represent the full data distribution. Despite this, it remains a simple and efficient approach widely used in practice (Figure 3).

#### 3.4.3. KDEF

The Karolinska Directed Emotional Faces (KDEF) dataset was developed by the Karolinska Institute in Sweden, primarily by researchers Daniel Lundqvist, Anders Flykt, and Arne Öhman. Introduced in 1998, KDEF contains 4900 images of 70 individuals (35 male, 35 female) displaying seven different emotional expressions: anger, disgust, fear, happiness, sadness, surprise, and neutral. Each emotion is presented in five different angles: full left profile, left, straight, right, and full right profile. The high-quality, standardized nature of the images makes KDEF an essential resource for studies in psychology and computer science, particularly in validating and training algorithms for and affective computing [31].

**Figure 4:**
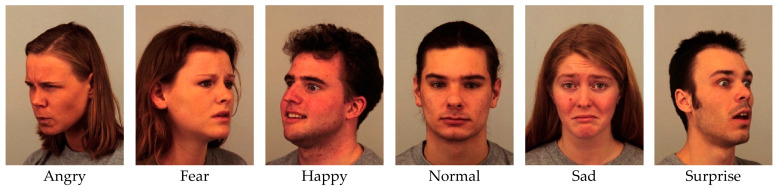
Example images from each class in the KDEF dataset.

In this study, only six emotion categories (anger, fear, happiness, sadness, surprise, and neutral) were used from the KDEF dataset, excluding the disgust class to ensure consistency with the selected experimental protocol and class alignment across datasets (Figure 4).

#### 3.4.4. JAFFE

The JAFFE (Japanese Female Facial Expression) dataset is a small yet widely utilized dataset for FER research. It contains 213 grayscale images of 10 Japanese female subjects, each portraying six basic emotions: anger, disgust, fear, happiness, sadness, and surprise, along with a neutral expression. Each expression is manually labeled, and the dataset is noted for its high-quality images with consistent lighting and frontal views.

**Figure 5:**
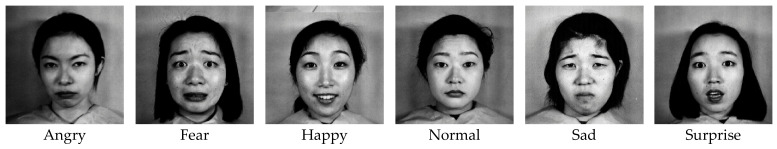
Example images from each class in the JAFFE dataset.

JAFFE also includes subjective ratings of emotional intensity for each image, enabling fine-tuned analysis. Despite its small size, JAFFE is a popular benchmark for testing algorithms due to its controlled conditions and clear expressions, making it suitable for initial exploration in emotion recognition and human–computer interaction studies Figure 5, [25].

### 3.5. Preprocessing

The FFM process is used to convert facial images into vectors suitable for training machine learning models. This involves a revised normalization technique executed in three key steps. First, facial landmarks are detected in each image. Second, these landmarks are extracted to form a structured representation.

**Figure 6:**
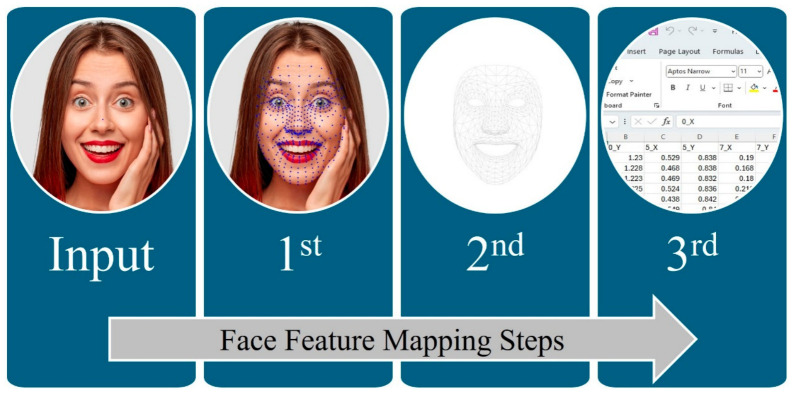
Architecture of FFM for images.

Third, normalization is applied to the extracted data, producing consistent feature values for model training. As shown in Figure 6, this pipeline ensures robust preprocessing of facial data. The paper used stratified k-fold (k = 4) cross-validation so that, in each fold, approximately 75% of the data were used for training and 25% for testing, enabling a closer comparison with the evaluation protocol adopted by Ciraolo et al.

### 3.6. Classification

Classification assigns input data to predefined categories based on learned patterns and is key to applications like image recognition and emotion detection. To evaluate this method, it was compared against four widely used classifiers: SVM, DT, RF, and MLP, selected for their prevalence in prior studies, varied learning strategies, and strong performance across classification tasks.

SVM: Support Vector Machines (SVM) are supervised learning models that construct optimal separating hyperplanes to distinguish between classes. They are effective in both binary and multi-class classification but can become computationally expensive with large-scale or high-dimensional data [5].

DT: Decision Trees (DT) classify data through a hierarchical structure of feature-based splits, where each internal node represents a decision rule, and each leaf node corresponds to a class label. They are simple and interpretable but prone to overfitting [7].

RF: Random Forests (RF) are ensemble methods that combine multiple decision trees trained on random subsets of data and features. Final predictions are obtained via majority voting, improving robustness and reducing overfitting compared to a single tree [8].

MLP: Multi-Layer Perceptrons (MLP) are feedforward neural networks composed of multiple fully connected layers. By learning non-linear mappings through backpropagation, they are well-suited for complex classification and regression tasks [9].

### 3.7. Evaluation

In FER, crucial evaluation metrics include accuracy, precision, recall and F1-score. Accuracy gauges the overall correctness of classifications, while precision and recall focus on specific positive instances. The F1-score strikes a balance between precision and recall, providing a comprehensive assessment of system performance. To enhance the selection process of the most suitable machine learning model, various tuning parameters have been applied, as outlined in Table 6.

In this paper, a total of 1020 tests were conducted to evaluate the effects of the feature list (L01–L15), the application of machine learning algorithms (SVM, DT, RF, MLP), and the tuning of specific hyperparameters. These comprehensive tests ensure an in-depth evaluation of the influence of feature list selection, algorithm choice, and hyperparameter optimization. The number of testing procedures follows the formula below for each algorithm:

L: Number of landmark groups (15 groups in total)

D: Number of datasets (4 datasets)

H: Number of primary hyperparameters associated with each ML algorithm

SH: Number of sub-hyperparameters corresponding to each primary hyperparameter (details in Table 6)

T: Total number of tests conducted for each ML algorithm

Based on these definitions, the total number of tests (TTT) for each ML algorithm was calculated as follows:

TSVM = 15 (L) × 4 (D) × 1 (H: Kernel) × 4 (SH) = 240

TDT = 15 (L) × 4 (D) × [1 (H: Criterion) × 3 (SH) + 1 (H: Splitter) × 2 (SH)] = 300

TRF = 15 (L) × 4 (D) × 1 (H: Criterion) × 3 (SH) = 180

TMLP = 15 (L) × 4 (D) × [1 (H: Solver) × 2 (SH) + 1 (H: Activation) × 3 (SH)] = 300 (As shown in Table 6, there are additional hyperparameters associated with MLP that are considered unique. Therefore, their specific count has not been included in the calculation of the total number of tests.)

These comprehensive tests ensure an in-depth evaluation of the influence of landmark group selection, algorithm choice, and hyperparameter optimization.

To ensure a statistically reliable evaluation, a k-fold cross-validation strategy was employed across all experiments. For each configuration, performance metrics including accuracy, precision, recall, and F1-score were computed over multiple folds, and their mean, standard deviation (std), and 95% confidence intervals (CI) were reported. This approach provides a more robust estimation of model generalization performance and reduces the risk of biased evaluation due to dataset partitioning. The inclusion of confidence intervals further allows assessing the stability and statistical significance of the obtained results.

In addition to descriptive statistical analysis, a McNemar’s test was conducted to perform pairwise comparisons between feature configurations, where the proposed FFM-L03 feature set was evaluated against L01 and L02 landmark groups. The comparison was carried out under the normalization framework described in this paper to ensure consistency across feature representations.

Moreover, the models used in the McNemar analysis were obtained through the hyperparameter tuning procedures defined in Table 6, and the evaluation was systematically performed across multiple configurations recorded in the experimental sheets. These configurations include dataset type, algorithm-specific hyperparameters (e.g., kernel in SVM, criterion and splitter in DT and RF, and solver and activation in MLP), as well as pairwise comparisons (L03 vs. L01 and L03 vs. L02). For each configuration, the corresponding evaluation metrics and statistical test outputs were recorded, enabling a structured and comprehensive comparison across different feature groups, classifiers, and tuned parameter settings.

The combination of cross-validation with statistical hypothesis testing strengthens the experimental methodology by providing both performance reliability (through mean, std, and CI) and statistical significance (through McNemar’s test).

This dual evaluation framework ensures that improvements attributed to the proposed feature mapping method (FFM-L03) are not only quantitatively higher but also statistically meaningful.

## 4. Results and Discussion

This section presents and discusses the experimental findings of the proposed facial expression recognition framework from multiple complementary perspectives.

First, the classification performance of the evaluated machine learning models is analyzed using standard metrics, including accuracy, precision, recall, and F1-score, across different datasets and feature mapping groups.

Second, the impact of feature selection and facial feature mapping strategies is examined to clarify how landmark distribution, subset design, and anatomical relevance influence recognition performance.

Third, the effect of the revised normalization method is assessed through comparative analyses against existing normalization strategies. In addition, statistical significance testing and runtime evaluation are included to provide a more comprehensive understanding of the proposed method in terms of robustness, fairness of comparison, and suitability for real-time deployment on resource-constrained edge platforms.

Table 7 presents a structured comparison between the proposed landmark-based framework and representative state-of-the-art CNN-based FER approaches, namely ASDC-FER and RepVGG.

The comparison highlights fundamental differences in input modality, computational requirements, and deployment assumptions. While CNN-based methods operate on high-dimensional RGB images and typically require GPU-level resources, the proposed approach relies on compact geometric landmark representations, enabling efficient inference on resource-constrained edge devices.

In addition, the proposed framework promotes privacy preservation and interpretability by avoiding raw image processing and utilizing explainable features. These distinctions indicate that the objective of this work is not to directly compete with accuracy-oriented SOTA models, but rather to address a complementary problem focused on real-time, low-cost, and privacy-aware FER suitable for embedded robotic applications. Therefore, the experimental results presented in the following sections should be interpreted within this application-driven context.

### 4.1. Feature Selection and Normalization Strategies

This subsection examines how different feature mapping groups and normalization strategies affect FER performance. The analysis focuses on the role of landmark subset design, spatial distribution, and revised normalization in shaping classification accuracy across datasets and models. By comparing empirical and mathematically derived feature groups, this section clarifies the trade-offs between accuracy, compactness, interpretability, and suitability for resource-constrained deployment.

#### 4.1.1. Accuracy Related to Feature Selection Procedure

**Figure 7:**
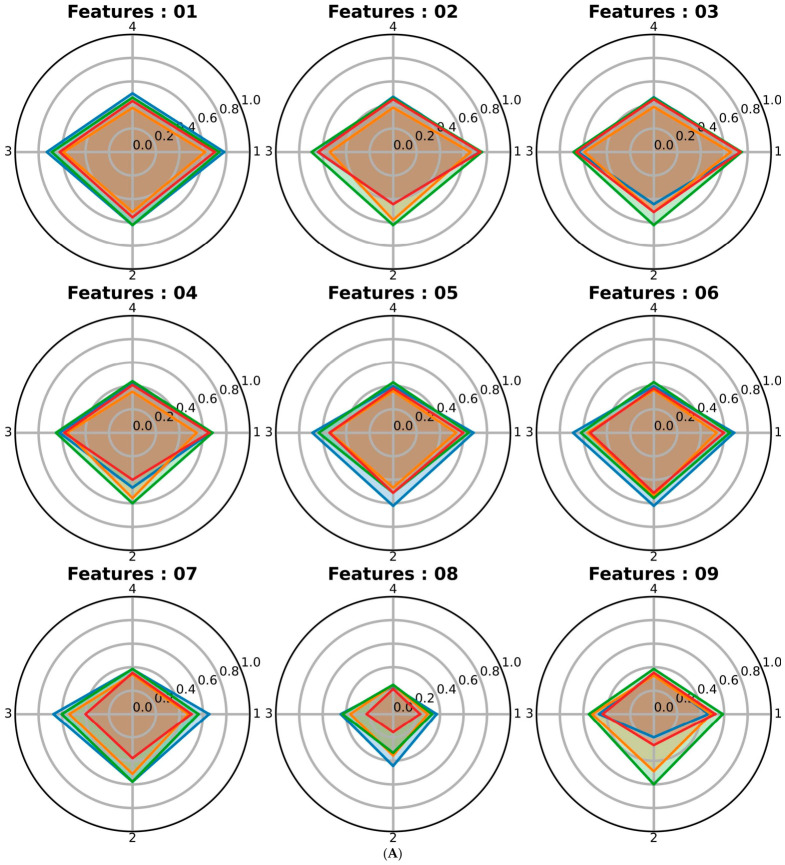

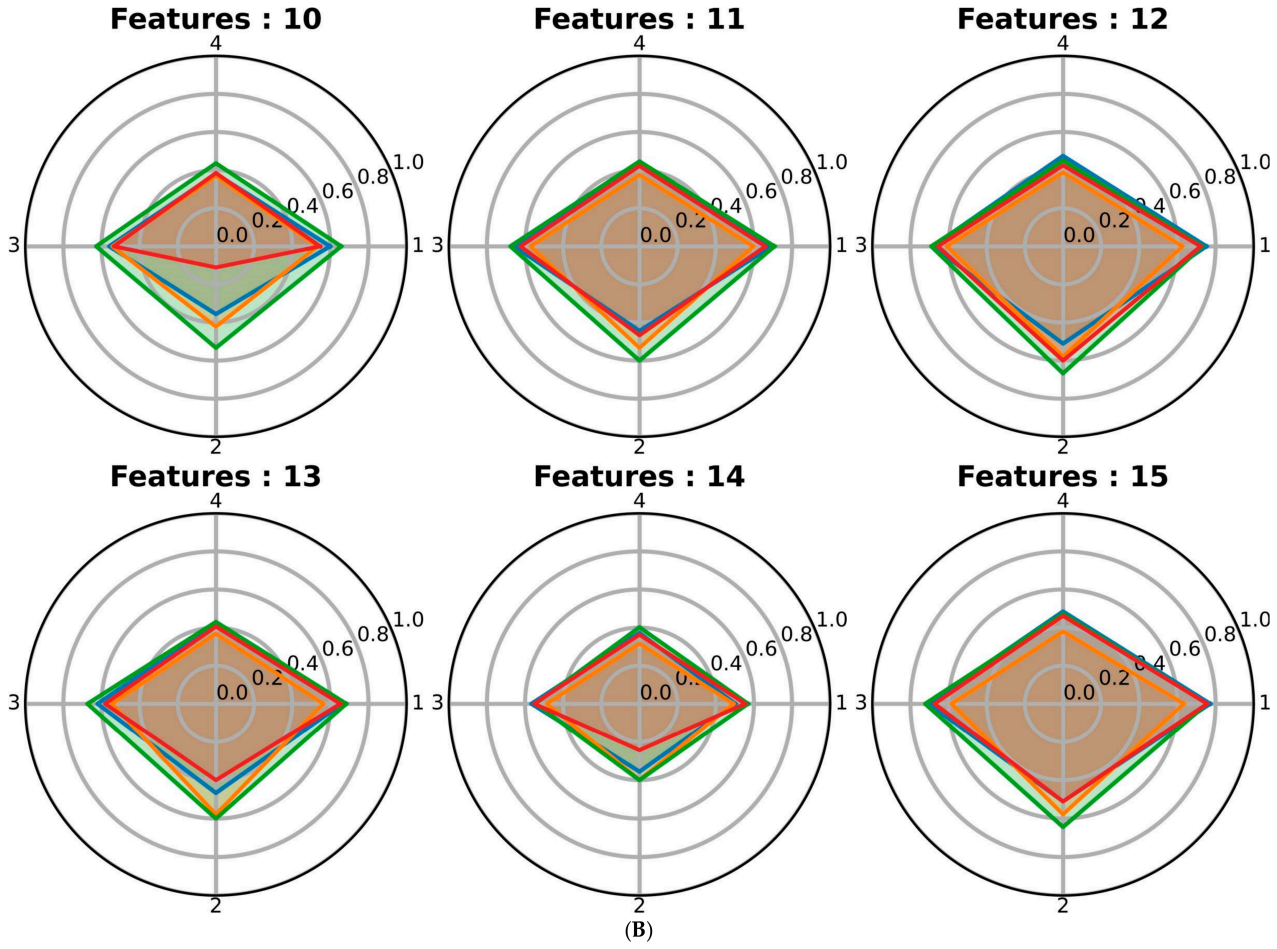
(**A**,**B**). Cumulative radar graphs based on Features groups (Table 3: L01–L15) and the maximum accuracy related to SVM (▬), DT (▬), RF (▬) and MLP (▬) for all applied data sets, FER 2013 (Axis no: 1), Freepik (Axis no: 2), JAFFE (Axis no: 3), KDEF (Axis no: 4). The values of each line in figure: 0.0, 0.2, 0.4, 0.6, 0.8, 1.0.

As part of the evaluation of features selected Figure 7A,B provides a cumulative radar graph representation of the maximum accuracy achieved by SVM, DT, RF, and MLP for all datasets: FER 2013 (Axis no: 1), Freepik (Axis no: 2), JAFFE (Axis no: 3), and KDEF (Axis no: 4). The radar plots enable a side-by-side comparison of feature configurations (Table 3: L01–L15) and their impact on classification performance. This figure emphasizes the relationship between feature selection methods, feature richness, and the resulting model accuracies, providing deeper insights into the effectiveness of statistical feature selection.

Given the large number of experiments conducted, radar charts were used to visualize the accuracy of each machine learning (ML) model across the different datasets, as shown in Figure 7A,B. Each radar chart corresponds to one of the FFM listed in Table 3; each polygon represents a classifier, and each vertex indicates its accuracy. In other words, Figure 7A,B summarizes the results of 240 accuracy experiments. To further improve the interpretability of the findings, Table 8 reports the maximum accuracy obtained by any ML model for each dataset, considering all hyperparameter combinations described in Table 6. Table 8 shows that L01 achieves the highest maximum accuracy on some datasets; however, the margin over L15 is small, and L15 even exceeds L01 on the Freepik dataset. This indicates that competitive FER performance can also be achieved with carefully selected subsets, and that landmark quality and spatial organization may be more important than simply using the full landmark set.

A direct comparison between L03 and L15 is particularly important. Although L15 achieves higher maximum accuracies than L03 in Table 8, it relies on a much larger subset and is derived from nonlinear manifold methods whose selected features are less anatomically structured and, in some cases, represented only in a single coordinate dimension. By contrast, L03 was designed as a compact and anatomically interpretable subset, guided by FACS-based priors and supported by multiple statistical criteria. Therefore, L15 offers strong predictive performance, whereas L03 provides a better balance between accuracy, compactness, interpretability, and suitability for resource-constrained robotic deployment.

However, the results indicate no direct correlation between the number of selected features and the obtained accuracy. For instance, the second-highest accuracy corresponds to feature group L15, which consists of 274 landmarks, with only a minor accuracy reduction of 0.009. Similarly, feature group L02, which includes only 60 landmarks (one-eighth of all available landmarks), results in an accuracy loss of just 0.026. A comparison of Table 8 with the landmark positions shown in Figure 1 highlights that the spatial distribution of landmarks plays a more significant role in FER than merely increasing the number of selected landmarks.

#### 4.1.2. Maximum Accuracy of Feature Selection Methods (L04–L15)

Table 8 summarizes the maximum classification accuracy achieved for each feature mapping group (FFM L01–L15) across all evaluated datasets (FER, Freepik, JAFFE, and KDEF). Each value represents the best-performing configuration obtained through different classifiers and their corresponding hyperparameter settings, providing an upper-bound estimate of performance for each feature group. This complementary perspective enables a direct comparison between empirically designed feature groups (L01–L03) and mathematically derived feature selection methods (L04–L15), facilitating the analysis of how landmark selection strategies influence the maximum attainable accuracy across datasets.

Analyzing feature groups L04–L15 (Table 8), which are derived from mathematical feature selection methods, along with their spatial distribution (Figure 1), reveals that empirical feature extraction for FFM should prioritize landmarks associated with the lips and chin. This observation led us to refine Ciraolo’s FFM by modifying L02 to L03 and adjusting the reference points of normalization to incorporate the influence of lower-face landmarks.

When comparing the performance of mathematical feature selection methods, the highest accuracy is achieved using the “t-SNE and UMAP” methods, which are more or less the same, regardless of the number of selected landmarks (see related FFM in Table 8). The next highest accuracy is obtained using the “ANOVA F-Score” method, specifically with 239 landmarks, approximately half of the total 478 landmarks. Analyzing feature groups L10, L11, and L12, which also utilize the “ANOVA F-Score” method, reveals a positive correlation between the number of selected landmarks and accuracy.

Results from the “Performance vs. Variance Threshold” method (FFM L04–L08) further support the idea that the spatial distribution of landmarks is more important than their quantity [32,33]. As shown in Table 8, accuracy improves from a threshold of 0.01 (241 landmarks) to 0.03 (93 landmarks) but declines when the threshold increases further, reducing the number of selected landmarks. The next strongest accuracy is obtained using the “LASSO” method with 241 landmarks, reinforcing the emphasis on landmark positioning rather than their total count.

Although the “Correlation Matrix of Features” and “PCA with 95% variance” methods, which select only 10 and 5 landmarks, respectively, achieve reasonable accuracy, they are generally not suitable for feature selection in FER. These findings highlight the importance of strategically selecting landmark positions to optimize FER performance.

A detailed examination of Table 8, where cells highlighting the highest achieved accuracy are marked in green, confirms that all feature selection methods, whether empirical or mathematically based, perform best in most FFMs with the FER 2013 dataset. This is primarily due to the significantly larger number of samples in the FER 2013 dataset compared to other datasets. The only exception is the “Performance vs. Variance Threshold” method, which demonstrates strong performance on the JAFFE dataset.

A direct comparison between L03 and L15 is particularly important. Although L15 achieves higher maximum accuracies than L03 in Table 8, it relies on a much larger subset and is derived from nonlinear manifold methods whose selected features are less anatomically structured and, in some cases, represented only in a single coordinate dimension. By contrast, L03 was designed as a compact and anatomically interpretable subset, guided by FACS-based priors and supported by multiple statistical criteria. Therefore, L15 offers strong predictive performance, whereas L03 provides a better balance between accuracy, compactness, interpretability, and suitability for resource-constrained robotic deployment.

Another important observation from Table 8 is the consistently low performance on KDEF, where maximum accuracy remains below 0.5 for all feature groups. This suggests that the evaluated models do not generalize well to this dataset. Possible reasons include the posed and highly standardized nature of KDEF expressions, limited sample diversity, differences in annotation style and expression intensity, and potential cultural or demographic mismatch relative to the other datasets. These factors may reduce the discriminative value of landmark-based features and make class separation more difficult, resulting in near-chance-level performance for some feature groups.

#### 4.1.3. Statistical Evaluation of Feature Mapping Groups

Table 9 presents the performance of different feature mapping groups (L01, L02, and L03) across four classifiers (SVM, DT, RF, and MLP) using standard evaluation metrics: accuracy, precision, recall, and F1-score. For each metric, three statistical indicators are reported: mean, standard deviation (std), and 95% confidence interval (CI-95%), computed via k-fold cross-validation.

Each row corresponds to a specific combination of feature group (FFM) and classifier, while the columns summarize both the central tendency (mean) and variability (std and CI) of model performance. The inclusion of standard deviation reflects the consistency of the model across folds, whereas the confidence interval provides an estimate of the reliability of the mean performance. This structure allows for a comprehensive comparison not only in terms of average performance but also in terms of stability and robustness across different experimental settings.

From Table 9, several observations can be made regarding the influence of feature groups and classifiers on performance.

First, SVM consistently achieves the highest accuracy values across all feature groups, particularly with L01 (0.775), indicating strong discriminative capability when combined with this feature representation. However, a slight decrease is observed for L02 and L03, suggesting that the effectiveness of SVM is sensitive to the selected feature group.

Second, RF demonstrates stable and competitive performance across all feature groups, with very close accuracy values (0.720–0.739) and relatively low standard deviations. Notably, RF achieves its best performance with L03 (0.739), indicating that the proposed feature mapping can be effectively exploited by ensemble-based methods.

Third, MLP shows moderate performance with noticeable variability, particularly for L01 and L03. Although its average accuracy is lower than SVM and RF, its performance remains consistent across metrics, suggesting that it can capture non-linear relationships but may require further tuning or architectural adjustments.

Regarding feature groups, L01 achieves the highest performance with SVM, while L03 shows competitive or slightly improved performance when combined with RF, suggesting that the proposed feature mapping (FFM-L03) may be more suitable for ensemble-based classifiers. Additionally, the relatively small standard deviations and narrow confidence intervals across most configurations indicate that the results are stable and reliable, reinforcing the validity of the evaluation framework.

#### 4.1.4. Comparison of Feature Mapping and Normalization (L01, L02, FFM-L03)

Table 10 presents a comparative evaluation of three feature mapping and normalization strategies (L01, L02, and the proposed FFM-L03) across multiple classifiers using accuracy, precision, recall, and F1-score. The reported values for FFM-L03 correspond to the averaged results obtained from the cross-validation analysis presented in Table 9, ensuring consistency with the statistical evaluation framework adopted in this study. In contrast, the results for L01 and L02 are derived from the normalization approaches introduced by Ciraolo et al., where L01 represents the full landmark configuration under their normalization method, and L02 corresponds to their empirically defined feature mapping strategy. The “Difference” columns quantify the absolute performance improvement achieved by FFM-L03 relative to L01 and L02, thereby highlighting the contribution of the proposed method. This unified presentation enables a clear comparison between prior normalization strategies and the proposed feature mapping approach under consistent evaluation criteria.

The comparative analysis highlights the performance differences between the traditional full map approach (L01) with Ciraolo’s empirical FFM and normalization method (L02) and FFM-L03, which is the proposed method for revision of normalization reference points in this paper. Across all classifiers (SVM, Decision Tree, Random Forest, and MLP), the L03 method demonstrates a consistent improvement in accuracy, precision, recall, and F1-scores over both L01 and L02. For instance, the accuracy for L03 with the SVM classifier reaches 0.741, compared to 0.532 for L01 and 0.526 for L02, as shown in Table 10. This indicates that the revised normalization reference points and FFM-L03 enhance the ability of the model to generalize and perform more reliably across datasets. When considering accuracy, L03 yields an average improvement of approximately 20% compared to L01 (e.g., 0.741 vs. 0.532 for SVM and 0.656 vs. 0.408 for Decision Tree) and an improvement of about 15% over L02 (e.g., 0.741 vs. 0.526 for SVM). Precision and recall exhibit similar trends, with L03 improving precision by up to 21% compared to L01 (e.g., 0.748 vs. 0.531 for SVM) and 17% over L02 (e.g., 0.748 vs. 0.525 for SVM). Recall improvements are in the range of 20% compared to L01 (e.g., 0.743 vs. 0.532 for SVM) and 17% over L02 (e.g., 0.743 vs. 0.526 for SVM). The observed F1-score improvements confirm these trends, with L03 achieving an F1-score of 0.745 for SVM, compared to 0.531 for L01 and 0.525 for L02, as detailed in Table 10.

The differences in performance metrics can be attributed to the methodological advancements in L03. Specifically, the refinement of normalization reference points appears to address key limitations in the earlier approaches by providing a more stable and consistent baseline for analysis. Additionally, FFM-L03 likely enhances feature extraction, contributing to the improved classification outcomes across all models.

### 4.2. Evaluation of Normalization

Table 11 presents a comparative evaluation of the Full Mapper (L01) under two different normalization strategies. The first main block, labeled Full Mapper (L01), based on Ciraolo et al.’s Normalization Method, reports the classification performance obtained using the original normalization approach introduced by Ciraolo et al. The second main block, labeled Full Mapper (L01) Using the Normalization Method in This Research, presents the corresponding performance achieved when applying the normalization method proposed in this study, where the reported values of the second main row represent the average evaluation metrics obtained from the experimental framework. The final block, labeled “Difference,” reports the absolute differences between the two normalization methods for each classifier (SVM, DT, RF, and MLP) across all evaluation metrics (accuracy, precision, recall, and F1-score), thereby providing a clear and structured quantification of the impact of the proposed normalization approach.

For example, the SVM classifier accuracy increases from 53.2% to 77.5%, a gain of 24.3%, accompanied by comparable improvements in precision (+24.7%), recall (+24.3%), and F1-score (+24.1%). Similar trends are observed for the other classifiers. The Decision Tree accuracy increases by 22.2%, while Random Forest and MLP show gains of 19.5% and 19.8%, respectively. These consistent improvements demonstrate the robustness of the revised normalization method and its ability to enhance the model’s overall discriminative capability.

These gains can be attributed to the methodological refinements introduced in the proposed normalization approach. By adjusting the reference geometry used for normalizing landmark coordinates, the revised method reduces distortions and yields a more stable and consistent coordinate system for feature extraction. This results in improved classifier generalization and higher accuracy across varied facial structures and configurations.

Table 12 presents a comparative evaluation of Ciraolo’s Face Mapper FFM (L02) under two different normalization strategies. The first main block, labeled Ciraolo’s Face Mapper FFM (L02) Based on Ciraolo et al.’s Normalization Method, reports the classification performance obtained using the original normalization approach introduced by Ciraolo et al. The second main block, labeled Ciraolo’s Face Mapper FFM (L02) Using the Normalization Method in This Research, presents the corresponding performance achieved when applying the normalization method proposed in this study, where the reported values represent the average evaluation metrics obtained from the experimental framework. The final block, labeled “Difference,” reports the absolute differences between the two normalization methods for each classifier (SVM, DT, RF, and MLP) across all evaluation metrics (accuracy, precision, recall, and F1-score), thereby providing a clear and structured quantification of the impact of the proposed normalization approach. For example, the SVM classifier shows a notable accuracy increase from 52.6% to 0.73.9%, a gain of 21.3%, accompanied by similar improvements in precision (+21.6%), recall (+21.3%), and F1-score (+20.7%). Comparable trends are observed across the remaining classifiers. The Decision Tree classifier yields an accuracy improvement of 20.8%, while the Random Forest and MLP classifiers show gains of 22.3% and 23.5%, respectively. These results underscore the robustness of the revised normalization method and its ability to enhance the discriminative performance of the L02 mapper across multiple learning models.

These improvements can be attributed to the methodological refinement introduced in the revised normalization approach. By replacing the upper-lip center with the nose center as the reference point, the method establishes a more stable and geometrically consistent facial coordinate system. This reduces distortions in the normalized landmark data and enables classifiers to better capture structural relationships among facial features, ultimately improving classification accuracy, stability, and generalizability.

### 4.3. Statistical Comparison of Feature Groups Using McNemar Analysis

Table 13, Table 14, Table 15 and Table 16 present the results of pairwise statistical comparisons between feature groups, specifically L03 vs. L01 and L03 vs. L02, across different classifiers and hyperparameter configurations. Each row corresponds to a unique experimental setting defined by the dataset, model-specific hyperparameters (e.g., kernel for SVM, criterion/splitter for DT and RF, and solver/activation for MLP), and the comparison pair.

For each configuration, the tables report the classification performance of L03 and the baseline feature group, the difference in accuracy, and the corresponding statistical test outputs, including the counts of disagreement cases. (b, c), the *p*-value, a significance indicator, and the performance outcome (improved, degraded, or no significant difference).

This structured representation enables a systematic evaluation of how the proposed feature group (L03) behaves relative to L01 and L02 under varying model configurations and datasets.

For Support Vector Machine (SVM) (Table 13), the results demonstrate a more diverse behavior across kernel functions. While some configurations (e.g., RBF and poly) lead to statistically significant improvements, others (e.g., linear and sigmoid) show degradation or no significant difference depending on the dataset.

This indicates that the effectiveness of L03 is strongly influenced by the choice of kernel, reflecting the dependency of SVM on the underlying feature space transformation.

For the Decision Tree (DT) classifier (Table 14), the results indicate that L03 generally leads to no statistically significant difference compared to L01 and L02 across most datasets and configurations. Only isolated cases, such as specific configurations in the FER dataset, show statistically significant degradation. This suggests that DT, as a single-tree model, has limited sensitivity to the differences between feature groups.

In the case of Random Forest (RF) (Table 15), a similar pattern is observed. Most configurations yield no significant difference, indicating that ensemble averaging reduces sensitivity to feature variations. However, a small number of cases (e.g., using the log_loss criterion with random splitting) show statistically significant degradation, suggesting that certain hyperparameter combinations may negatively interact with the proposed feature representation.

The Multi-Layer Perceptron (MLP) results (Table 16) further emphasize the role of hyperparameters. Models using the Adam solver tend to show no significant difference, indicating stable behavior across feature groups. In contrast, configurations using SGD exhibit several statistically significant changes, including both improvements and degradations, depending on the dataset and comparison baseline.

This suggests that optimization dynamics and convergence behavior play a critical role in how feature representations are utilized.

Overall, these findings demonstrate that hyperparameter tuning is a key factor in evaluating feature representations, as it directly influences the interaction between the classifier and the feature space. While the proposed feature group (L03) shows competitive performance across many configurations, its relative advantage or disadvantage is not uniform and depends on the learning model and parameter settings. This reinforces the need for a comprehensive evaluation framework that considers both model selection and hyperparameter optimization when assessing feature engineering methods.

### 4.4. Runtime Performance Evaluation for Edge Deployment

To further substantiate the lightweight nature of the proposed framework and its suitability for edge-based deployment, a detailed runtime analysis was conducted on representative low-resource hardware. Experiments were performed on a Raspberry Pi 3 Model B+, equipped with a 1.4 GHz 64-bit quad-core processor and limited memory capacity, reflecting realistic edge-computing constraints. The proposed FFM-L03 method achieved an average processing speed of 9.59 FPS (≈104 ms per frame, computed over the last 60 detections), demonstrating near real-time performance under constrained conditions. For comparison, a lightweight MobileNet (mini) model achieved 11.72 FPS (≈85 ms per frame), while a larger MobileNet variant achieved 8.17 FPS (≈122 ms per frame). These results highlight the trade-off between model complexity and inference speed, where compact deep models may offer higher throughput at the cost of increased memory footprint, while larger architectures incur additional computational overhead. In contrast, the proposed approach relies on a reduced and discriminative landmark representation, leading to lower computational and memory requirements while maintaining competitive performance (Table 17). For reproducibility and to facilitate further comparative experiments, the implementation details, trained models, and evaluation scripts are made publicly available: https://github.com/hosamzolfonoon/Papep_FER_Test (accessed on 25 January 2025). 

## 5. Conclusions

This paper presented a lightweight vision-based FER framework for assistive healthcare robotics based on stable landmark normalization and compact facial feature mapping. The study showed that FER performance is influenced not only by the number of landmarks but more importantly by their spatial distribution, anatomical relevance, and geometric normalization. To address these issues, the paper introduced a revised nose-centered normalization strategy and the proposed FFM-L03, which combines FACS-guided anatomical priors with multiple analytical selection methods to retain 60 informative landmarks.

The experimental results across multiple datasets, feature groups, classifiers, and statistical comparisons demonstrate that the proposed framework provides a strong balance between recognition performance, compactness, interpretability, and deployment efficiency. In particular, the revised normalization and FFM-L03 consistently outperformed both the full-landmark configuration and the Ciraolo baseline, with improvements of up to 22.1 percentage points in accuracy, precision, recall, and F1-score under several settings. Runtime analysis on Raspberry Pi-class hardware further showed that the method supports near real-time execution, confirming its suitability for low-power robotic platforms.

The importance of this work lies in showing that privacy-preserving and resource-aware FER can be improved substantially without relying on computationally expensive deep image-based models. By operating on anonymized landmark coordinates and avoiding raw image storage or transmission, the proposed framework is especially relevant for sensitive domains such as elderly care and telepresence healthcare.

Future work will focus on cross-dataset and cross-domain generalization, multi-annotator validation of custom datasets, temporal modeling of facial dynamics, and in situ evaluation on real assistive robotic platforms operating in unconstrained environments. These directions will help further assess robustness, scalability, and practical readiness for real-world deployment.

## Figures and Tables

**Table 1:** Summary Table of Related Work (FER and Telepresence Robotics).

Short Title	Methods	Finding/Main Result	Year	Ref
Emotional AI for Tele-Rehabilitation	FER pipeline using MediaPipe + ML classifiers; Emotional AI framework	Demonstrated reliable real-time emotion monitoring to support remote therapy and clinician awareness	2024	[9]
Pudu Robot for Emotional Care in COVID-19 ICUs	Telepresence robot with audio-visual interaction (no deep FER focus)	Enabled safe patient-staff interaction and emotional support during COVID-1 learning context using deep learning models 9, reducing infection risk	2021	[11]
GDPR Compliance in Telemedicine	Legal and regulatory review	Identified compliance challenges and requirements for telemedicine systems handling sensitive data	2023	[12]
FER Methods Review	Survey of traditional ML, CNNs, RNNs, and datasets	Concluded that deep learning significantly improves FER accuracy but depends on data quality	2019	[13]
Telepresence Social Robotics Review	Survey of telepresence robot systems and interaction models	Emphasized the importance of social cues (including facial expressions) for co-presence	2022	[14]
Affective Telepresence Robot (Haru)	MobileNet-based CNN for FER + reinforcement learning for behavior selection	Robot improved affect-aware responses over time, reducing the tele-operator workload	2021	[15]
FER-Based Learner Engagement Detection	MediaPipe Face Mesh (≈470 landmarks) + deep neural networks	Improved emotion and engagement detection accuracy in online learning environments	2023	[16]
Masked Face Emotion Recognition	Facial landmark extraction + deep learning	Demonstrated robust FER even with face masks, useful for real-time applications	2023	[17]
Telepresence Robots in Healthcare	Review of telepresence robots and control techniques	Confirmed effectiveness in remote consultations and infection control	2022	[18]
Healthcare Robotic Telepresence	System-level review of THRs	Identified benefits for remote monitoring and consultations in healthcare settings	2021	[19]
Testing Telepresence Robots in Healthcare	Empirical testing scenarios and user studies	Found workload reduction, but noted usability and acceptance challenges	2024	[20]
Telepresence Robots for Special Needs	Systematic review	Highlighted accessibility and usability barriers; called for inclusive design improvements	2022	[21]
Telepresence Robots in Aged Care	Scoping review protocol	Identified potential for reducing loneliness via family connectivity, especially during pandemics	2021	[22]

**Table 2:** Anatomical Interpretation of Feature Sets Defined in Table.

FFM List	Spatial Behavior	Main Facial Regions Emphasized	Anatomical Meaning (FACS)	Interpretation for FER
L01	Uniform, dense coverage	Entire face (including stable regions)	Includes all action units, but without prioritization	Redundant representation; no anatomical selectivity
L02	Structured predefined subset	Mouth, eyes, eyebrows	Targets key action units based on prior FER design	Anatomically meaningful but fixed and non-adaptive
L03	Balanced and coherent distribution	Mouth, chin, eyes, eyebrows, cheeks	Strong alignment with AU12, AU15, AU17, AU25, AU26	Optimal balance between anatomical relevance and compactness
L04–L08	Increasing spatial concentration	Lower face (mouth, lips, chin, jawline)	Emphasizes high-mobility AUs (AU12, AU25, AU26, AU17)	Captures dynamic regions but may omit stable expressive cues
L09	Sparse and fragmented	No consistent regional focus	Weak mapping to coherent action-unit regions	Limited anatomical interpretability
L10–L12	Regionally clustered and structured	Mouth, eyes, eyebrows, cheeks	Strong correspondence to both upper and lower face AUs	High discriminative and anatomical relevance
L13	Compact and grouped	Mouth, chin, nasolabial area	Preserves coordinated AU interactions	Efficient and anatomically consistent subset
L14	Highly compressed, central	Central face (nose region)	Poor correspondence to expressive AUs	Low anatomical interpretability
L15	Broad and dense distribution	Eyes, eyebrows, cheeks, mouth, chin	Covers multiple AU regions across the face	High coverage but less structured representation

**Table 3:** List of features, selection methods applied, and the number of features selected counts.

FFM List	Method	Number of Selected Features
L01	All landmarks	478
L02	Ciraolo’s Feature Mapper	60
L03	Empirical Mapper method of this research	60
L04	Performance vs. Variance Threshold = 0.01	241
L05	Performance vs. Variance Threshold = 0.02	142
L06	Performance vs. Variance Threshold = 0.03	93
L07	Performance vs. Variance Threshold = 0.04	52
L08	Performance vs. Variance Threshold = 0.05	29
L09	Correlation Matrix of Features	10
L10	Anova F-score for Feature Selection 119/2 = 59	59
L11	Anova F-score for Feature Selection 239/2 = 119	119
L12	Anova F-score for Feature Selection 487/2 = 239	239
L13	LASSO	241
L14	PCA with 95% variance	5
L15	tSNE and UMAP	274

**Table 4:** Anatomical grouping and functional role of selected facial landmarks in FFM-L03.

Facial Region	Landmark Indices	Functional Role in FER	Related FACS Action Units
Eyebrows	70, 63, 105, 66, 107, 55, 300, 293, 334, 296, 336, 285	Capture brow movement and tension associated with emotional intensity	AU1, AU2, AU4
Eyelid/ Eye Opening	159, 145, 263, 362	Encode eye openness, blinking, and alertness	AU5, AU7
Cheeks/ Smile Lines	118, 50, 347, 280, 216, 436	Represent cheek raising and nasolabial deformation during smiling	AU6, AU12
Mouth/ Lip Core	0, 11, 13, 14, 17, 61, 291, 84, 314, 83, 18, 313	Capture primary expression dynamics (smile, frown, mouth opening)	AU12, AU15, AU25, AU26
Chin/ Lower Face	171, 175, 396, 148, 152, 377, 400, 176, 378, 149	Reflect lower-face tension and deformation	AU17
Lower Facial Boundary	369, 140, 262, 32	Provide structural support for facial contour and normalization stability	-
Normalization Anchors	10, 5, 454, 234	Used as stable geometric references for alignment	-
Iris	468, 473	Capture gaze direction and subtle eye dynamics	-

**Table 5:** Number of samples for all emotions in all original datasets.

Dataset	Angry	Fear	Happy	Normal	Sad	Surprise	Sum
FER-2013	4958	5121	8989	6198	6077	4002	35,345
Freepik	200	200	200	200	200	200	1400
JAFFE	30	32	31	30	31	30	184
KDEF	700	700	700	700	700	700	4200

**Table 6:** List of parameters which were applied in model tunning procedure.

ML Algorithms	Parameters Applied in Model Tuning
SVM	Kernel = [‘linear’, ‘poly’, ‘rbf’, ‘sigmoid’]
DT	Criterion = [‘gini’, ‘entropy’, ‘log_loss’] Splitter = [‘best’, ‘random’]
RF	Criterion = [‘gini’, ‘entropy’, ‘log_loss’]
MLP	Solver = [‘sgd’, ‘adam’], Activation = [‘logistic’, ‘tanh’, ‘relu’], Number of hidden_layer = [100] Iteration = [200], Learning rate = [0.001]

**Table 7:** Comparative analysis of the proposed method and representative SOTA FER approaches.

Aspect	Proposed Method (L03—Landmark-Based)	ASDC-FER (SOTA CNN-Based)	RepVGG (SOTA CNN-Based)
Input Modality	Facial landmarks (geometric features)	RGB facial images	RGB facial images
Model Type	Lightweight ML (SVM, RF, DT, MLP)	Deep CNN with attention mechanisms	Deep CNN (VGG-style re-parameterization)
Computational Cost	Low (CPU-friendly)	High (GPU required)	High (GPU required)
Hardware Target	Edge devices (e.g., Raspberry Pi, robots)	High-performance GPUs	High-performance GPUs
Training Requirements	Small to medium datasets	Large-scale datasets	Large-scale datasets
Inference Speed	Real-time on embedded systems	Limited to edge devices	Limited to edge devices
Privacy	High (no image storage required)	Low (raw images processed)	Low (raw images processed)
Design Goal	Efficiency, robustness, explainability	Maximum accuracy	Maximum accuracy
Suitability for Robotics	High (telepresence/healthcare robots)	Limited	Limited
Explainability	High (interpretable features)	Low (black-box CNN)	Low (black-box CNN)
Benchmark Role in This Study	Primary focus	Not directly comparable (different paradigm)	Not directly comparable

**Table 8:** Maximum accuracy achieved for each applied dataset regarding the FFM list.

FFM List	FER	Freepik	JAFFE	KDEF
	Max	Max	Max	Max
L01	0.781	0.622	0.730	0.499
L02	0.755	0.622	0.695	0.470
L03	0.748	0.622	0.684	0.466
L04	0.681	0.600	0.652	0.439
L05	0.684	0.622	0.688	0.429
L06	0.684	0.622	0.688	0.431
L07	0.653	0.578	0.674	0.385
L08	0.371	0.444	0.447	0.250
L09	0.588	0.600	0.553	0.385
L10	0.657	0.533	0.628	0.436
L11	0.708	0.600	0.677	0.446
L12	0.752	0.667	0.691	0.475
L13	0.684	0.600	0.674	0.430
L14	0.569	0.400	0.567	0.402
L15	0.772	0.644	0.723	0.485

**Table 9:** Performance comparison of feature mapping groups (L01–L03) across classifiers using k-fold cross-validation (mean, standard deviation, and 95% confidence interval).

FFM	Classifier	Accuracy			Precision			Recall			F1-Score		
		Mean	Std	CI-%95	Mean	Std	CI-%95	Mean	Std	CI-%95	Mean	Std	CI-%95
L01	SVM	0.775	0.022	0.022	0.778	0.024	0.024	0.775	0.022	0.022	0.772	0.023	0.023
	DT	0.630	0.027	0.026	0.632	0.024	0.024	0.630	0.027	0.026	0.629	0.026	0.025
	RF	0.720	0.020	0.020	0.719	0.023	0.023	0.720	0.020	0.020	0.717	0.022	0.022
	MLP	0.708	0.028	0.027	0.712	0.027	0.026	0.708	0.028	0.027	0.705	0.026	0.025
L02	SVM	0.739	0.013	0.013	0.741	0.014	0.014	0.739	0.013	0.013	0.732	0.013	0.013
	DT	0.629	0.020	0.020	0.630	0.021	0.021	0.629	0.020	0.020	0.628	0.021	0.021
	RF	0.738	0.018	0.018	0.737	0.018	0.018	0.738	0.018	0.018	0.735	0.018	0.018
	MLP	0.727	0.021	0.021	0.725	0.022	0.022	0.727	0.021	0.021	0.723	0.021	0.021
L03	SVM	0.733	0.013	0.013	0.736	0.016	0.016	0.733	0.013	0.013	0.726	0.014	0.014
	DT	0.629	0.033	0.032	0.629	0.034	0.033	0.629	0.033	0.032	0.628	0.034	0.033
	RF	0.739	0.017	0.017	0.738	0.016	0.016	0.739	0.017	0.017	0.737	0.016	0.016
	MLP	0.714	0.025	0.024	0.713	0.027	0.026	0.714	0.025	0.024	0.710	0.026	0.025

**Table 10:** Performance Comparison of FFM and Normalization Methods (L01, L02, L03) Across Classification Models.

FFM	Classifier	Accuracy	Precision	Recall	F1-Score	Difference Accuracy	Difference Precision	Difference Recall	Difference F1-Score
Full (L01)	SVM	0.532	0.531	0.532	0.531	0.201	0.205	0.201	0.195
	DT	0.408	0.413	0.408	0.410	0.221	0.216	0.221	0.218
	RF	0.525	0.521	0.525	0.521	0.214	0.217	0.214	0.216
	MLP	0.510	0.504	0.510	0.498	0.204	0.209	0.204	0.212
Ciraolo (L02)	SVM	0.526	0.525	0.526	0.525	0.207	0.211	0.207	0.201
	DT	0.421	0.419	0.421	0.419	0.208	0.21	0.208	0.209
	RF	0.515	0.510	0.515	0.510	0.224	0.228	0.224	0.227
	MLP	0.492	0.486	0.492	0.478	0.222	0.227	0.222	0.232
FFM-L03	SVM	0.733	0.736	0.733	0.726	-	-	-	-
	DT	0.629	0.629	0.629	0.628	-	-	-	-
	RF	0.739	0.738	0.739	0.737	-	-	-	-
	MLP	0.714	0.713	0.714	0.710	-	-	-	-

**Table 11:** Comparison of Classification Performance Metrics Using Ciraolo’s and Revised Normalization Methods.

FFM	Classifier	Accuracy	Precision	Recall	F1-Score
Full Mapper (L01) Based on Ciraolo et al.’s Normalization Method	SVM	0.532	0.531	0.532	0.531
	DT	0.408	0.413	0.408	0.410
	RF	0.525	0.521	0.525	0.521
	MLP	0.510	0.504	0.510	0.498
Full Mapper (L01) Using the Normalization Method in This Research	SVM	0.775	0.778	0.775	0.772
	DT	0.630	0.632	0.630	0.629
	RF	0.720	0.719	0.720	0.717
	MLP	0.708	0.712	0.708	0.705
Difference	SVM	0.243	0.247	0.243	0.241
	DT	0.222	0.219	0.222	0.219
	RF	0.195	0.198	0.195	0.196
	MLP	0.198	0.208	0.198	0.207

**Table 12:** Comparison of Classification Metrics for FFM (L02) Using Ciraolo’s and Revised Normalization Methods.

FFM	Classifier	Accuracy	Precision	Recall	F1-Score
Ciraolo’s FFM (L02) Based on Ciraolo et al.’s Normalization Method	SVM	0.526	0.525	0.526	0.525
	DT	0.421	0.419	0.421	0.419
	RF	0.515	0.510	0.515	0.510
	MLP	0.492	0.486	0.492	0.478
Ciraolo’s FFM(L02) Using the Normalization Method in This Research	SVM	0.739	0.741	0.739	0.732
	DT	0.629	0.630	0.629	0.628
	RF	0.738	0.737	0.738	0.735
	MLP	0.727	0.725	0.727	0.723
Difference	SVM	0.213	0.216	0.213	0.207
	DT	0.208	0.211	0.208	0.209
	RF	0.223	0.227	0.223	0.225
	MLP	0.235	0.239	0.235	0.245

**Table 13:** McNemar Test Results for Support Vector Machine (SVM) with different kernel functions “No” indicates no statistically significant difference.

Comparison (L03 vs. Baseline)	Dataset	Kernel Function	Accuracy (FFM-L03)	Accuracy (Baseline)	Δ Accuracy	Discordant Pairs (b)	Discordant Pairs (c)	*p*-Value	Performance Outcome
L01	FER	linear	0.473	0.505	−0.031	251	410	0.000000	Degraded
		poly	0.430	0.419	0.011	179	122	0.001211	Improved
		rbf	0.421	0.413	0.008	168	125	0.014003	Improved
		sigmoid	0.235	0.259	−0.024	187	311	0.000000	Degraded
	Freepik	linear	0.672	0.741	−0.069	39	117	0.000000	Degraded
		poly	0.563	0.543	0.020	76	54	0.065086	No
		rbf	0.536	0.507	0.029	79	46	0.004025	Improved
		sigmoid	0.394	0.409	−0.015	86	103	0.244421	No
	JAFFE	linear	0.506	0.611	−0.106	9	28	0.002563	Degraded
		poly	0.294	0.311	−0.017	3	6	0.507813	No
		rbf	0.306	0.317	−0.011	1	3	0.625000	No
		sigmoid	0.294	0.294	0.000	3	3	1.000000	No
	KDEF	linear	0.733	0.775	−0.042	69	177	0.000000	Degraded
		poly	0.632	0.598	0.035	177	89	0.000000	Improved
		rbf	0.605	0.540	0.064	276	113	0.000000	Improved
		sigmoid	0.274	0.268	0.006	83	68	0.254500	No
L02	FER	linear	0.473	0.473	0.001	152	148	0.862527	No
		poly	0.430	0.436	−0.005	141	169	0.125016	No
		rbf	0.421	0.427	−0.006	125	155	0.082897	No
		sigmoid	0.235	0.216	0.018	267	173	0.000009	Improved
	Freepik	linear	0.672	0.668	0.004	33	29	0.703537	No
		poly	0.563	0.570	−0.007	35	43	0.428207	No
		rbf	0.536	0.529	0.007	50	42	0.465707	No
		sigmoid	0.394	0.436	−0.043	36	84	0.000014	Degraded
	JAFFE	linear	0.506	0.494	0.011	6	4	0.753906	No
		poly	0.294	0.322	−0.028	2	7	0.179688	No
		rbf	0.306	0.306	0.000	3	3	1.000000	No
		sigmoid	0.294	0.306	−0.011	2	4	0.687500	No
	KDEF	linear	0.733	0.739	−0.006	53	69	0.174205	No
		poly	0.632	0.631	0.002	74	69	0.738137	No
		rbf	0.605	0.618	−0.013	67	101	0.010679	Degraded
		sigmoid	0.274	0.274	0.000	89	88	1.000000	No

**Table 14:** McNemar Test Results for Decision Tree (DT) across datasets and hyperparameter configurations. “No” indicates no statistically significant difference.

Comparison (L03 vs. Baseline)	Dataset	Criterion Function	Splitter Function	Accuracy (FFM-L03)	Accuracy (Baseline)	Δ Accuracy	Discordant Pairs (b)	Discordant Pairs (c)	*p*-Value	Performance Outcome
L01	FER	entropy	best	0.377	0.369	0.008	823	783	0.330467	No
			random	0.372	0.372	0.000	817	815	0.980252	No
		gini	best	0.376	0.375	0.001	793	787	0.899904	No
			random	0.367	0.362	0.005	803	779	0.563101	No
		log_loss	best	0.375	0.362	0.013	847	781	0.107159	No
			random	0.356	0.369	−0.013	757	825	0.092053	No
	Freepik	entropy	best	0.572	0.540	0.032	201	165	0.067181	No
			random	0.548	0.560	−0.012	198	212	0.520908	No
		gini	best	0.548	0.548	0.000	168	168	1.000000	No
			random	0.550	0.574	−0.024	192	219	0.199614	No
		log_loss	best	0.568	0.562	0.006	193	186	0.757978	No
			random	0.557	0.575	−0.019	202	223	0.331981	No
	JAFFE	entropy	best	0.589	0.589	0.000	28	28	1.000000	No
			random	0.539	0.494	0.044	37	29	0.389052	No
		gini	best	0.617	0.572	0.044	28	20	0.312327	No
			random	0.550	0.561	−0.011	34	36	0.904975	No
		log_loss	best	0.600	0.572	0.028	31	26	0.596642	No
			random	0.511	0.572	−0.061	28	39	0.221549	No
	KDEF	entropy	best	0.629	0.630	−0.001	387	390	0.942807	No
			random	0.605	0.593	0.011	464	435	0.350386	No
		gini	best	0.614	0.603	0.011	404	376	0.333670	No
			random	0.595	0.602	−0.007	434	452	0.567939	No
		log_loss	best	0.619	0.624	−0.005	377	389	0.691065	No
			random	0.601	0.607	−0.006	455	470	0.645312	No
L02	FER	entropy	best	0.377	0.367	0.010	751	701	0.198455	No
			random	0.372	0.365	0.007	831	795	0.385413	No
		gini	best	0.376	0.376	0.001	718	715	0.957867	No
			random	0.367	0.378	−0.011	804	859	0.185426	No
		log_loss	best	0.375	0.367	0.008	739	698	0.291334	No
			random	0.356	0.377	−0.021	759	865	0.009152	Degraded
	Freepik	entropy	best	0.572	0.569	0.003	170	167	0.913266	No
			random	0.548	0.563	−0.015	201	218	0.434455	No
		gini	best	0.548	0.554	−0.006	137	144	0.720465	No
			random	0.550	0.547	0.003	213	210	0.922548	No
		log_loss	best	0.568	0.562	0.006	172	165	0.743843	No
			random	0.557	0.553	0.004	204	200	0.881376	No
	JAFFE	entropy	best	0.589	0.539	0.050	35	26	0.305677	No
			random	0.539	0.500	0.039	32	25	0.427043	No
		gini	best	0.617	0.578	0.039	29	22	0.401062	No
			random	0.550	0.544	0.006	32	31	1.000000	No
		log_loss	best	0.600	0.528	0.072	35	22	0.111161	No
			random	0.511	0.528	−0.017	39	42	0.824313	No
	KDEF	entropy	best	0.629	0.629	0.000	278	279	1.000000	No
			random	0.605	0.595	0.010	484	459	0.434496	No
		gini	best	0.614	0.607	0.007	325	308	0.524846	No
			random	0.595	0.589	0.006	461	445	0.618268	No
		log_loss	best	0.619	0.627	−0.008	258	278	0.411855	No
			random	0.601	0.594	0.007	479	460	0.556953	No

**Table 15:** McNemar Test Results for Random Forest (RF) under different splitting criteria. “No” indicates no statistically significant difference.

Comparison (L03 vs. Baseline)	Dataset	Kernel Function	Accuracy (FFM-L03)	Accuracy (Baseline)	Δ Accuracy	Discordant Pairs (b)	Discordant Pairs (c)	*p*-Value	Performance Outcome
L01	FER	entropy	0.477	0.472	0.005	369	342	0.329525	No
		gini	0.472	0.475	−0.004	349	367	0.525249	No
		log_loss	0.477	0.472	0.005	369	342	0.329525	No
	Freepik	entropy	0.689	0.692	−0.004	62	66	0.791007	No
		gini	0.675	0.678	−0.004	58	62	0.784328	No
		log_loss	0.689	0.692	−0.004	62	66	0.791007	No
	JAFFE	entropy	0.728	0.717	0.011	10	8	0.814529	No
		gini	0.733	0.739	−0.006	8	9	1.000000	No
		log_loss	0.728	0.717	0.011	10	8	0.814529	No
	KDEF	entropy	0.739	0.720	0.019	148	100	0.002767	Improved
		gini	0.734	0.714	0.020	145	93	0.000908	Improved
		log_loss	0.739	0.720	0.019	148	100	0.002767	Improved
L02	FER	entropy	0.477	0.474	0.003	360	345	0.598037	No
		gini	0.472	0.483	−0.011	328	385	0.035901	Degraded
		log_loss	0.477	0.474	0.003	360	345	0.598037	No
	Freepik	entropy	0.689	0.698	−0.009	48	58	0.382126	No
		gini	0.675	0.690	−0.015	44	61	0.118000	No
		log_loss	0.689	0.698	−0.009	48	58	0.382126	No
	JAFFE	entropy	0.728	0.700	0.028	13	8	0.383310	No
		gini	0.733	0.717	0.017	11	8	0.647606	No
		log_loss	0.728	0.700	0.028	13	8	0.383310	No
	KDEF	entropy	0.739	0.738	0.000	113	112	1.000000	No
		gini	0.734	0.737	−0.002	101	107	0.728919	No
		log_loss	0.739	0.738	0.000	113	112	1.000000	No

**Table 16:** McNemar Test Results for Multi-Layer Perceptron (MLP) under different solver and activation settings (hidden layer size = 100, number of iterations = 200, learning rate = 0.001). “No” indicates no statistically significant difference.

Comparison (L03 vs. Baseline)	Dataset	Solver Function	Activation Function	Accuracy (FFM-L03)	Accuracy (Baseline)	Δ Accuracy	Discordant Pairs (b)	Discordant Pairs (c)	*p*-Value	Performance Outcome
L01	FER	adam	logistic	0.473	0.465	0.008	462	420	0.167384	No
			relu	0.473	0.465	0.008	462	420	0.167384	No
			tanh	0.473	0.465	0.008	462	420	0.167384	No
		sgd	logistic	0.411	0.436	−0.025	270	398	0.000001	Degraded
			relu	0.411	0.436	−0.025	270	398	0.000001	Degraded
			tanh	0.411	0.436	−0.025	270	398	0.000001	Degraded
	Freepik	adam	logistic	0.637	0.667	−0.030	76	110	0.015309	Degraded
			relu	0.637	0.667	−0.030	76	110	0.015309	Degraded
			tanh	0.637	0.667	−0.030	76	110	0.015309	Degraded
		sgd	logistic	0.495	0.528	−0.034	110	148	0.021069	Degraded
			relu	0.495	0.528	−0.034	110	148	0.021069	Degraded
			tanh	0.495	0.528	−0.034	110	148	0.021069	Degraded
	JAFFE	adam	logistic	0.511	0.594	−0.083	6	21	0.005925	Degraded
			relu	0.511	0.594	−0.083	6	21	0.005925	Degraded
			tanh	0.511	0.594	−0.083	6	21	0.005925	Degraded
		sgd	logistic	0.211	0.433	−0.222	13	53	0.000001	Degraded
			relu	0.211	0.433	−0.222	13	53	0.000001	Degraded
			tanh	0.211	0.433	−0.222	13	53	0.000001	Degraded
	KDEF	adam	logistic	0.714	0.708	0.006	153	138	0.411865	No
			relu	0.714	0.708	0.006	153	138	0.411865	No
			tanh	0.714	0.708	0.006	153	138	0.411865	No
		sgd	logistic	0.515	0.599	−0.084	80	293	0.000000	Degraded
			relu	0.515	0.599	−0.084	80	293	0.000000	Degraded
			tanh	0.515	0.599	−0.084	80	293	0.000000	Degraded
L02	FER	adam	logistic	0.473	0.467	0.006	248	218	0.179080	No
			relu	0.473	0.467	0.006	248	218	0.179080	No
			tanh	0.473	0.467	0.006	248	218	0.179080	No
		sgd	logistic	0.411	0.405	0.006	264	234	0.193714	No
			relu	0.411	0.405	0.006	264	234	0.193714	No
			tanh	0.411	0.405	0.006	264	234	0.193714	No
	Freepik	adam	logistic	0.637	0.647	−0.011	32	44	0.206737	No
			relu	0.637	0.647	−0.011	32	44	0.206737	No
			tanh	0.637	0.647	−0.011	32	44	0.206737	No
		sgd	logistic	0.495	0.459	0.035	85	45	0.000572	Improved
			relu	0.495	0.459	0.035	85	45	0.000572	Improved
			tanh	0.495	0.459	0.035	85	45	0.000572	Improved
	JAFFE	adam	logistic	0.511	0.517	−0.006	8	9	1.000000	No
			relu	0.511	0.517	−0.006	8	9	1.000000	No
			tanh	0.511	0.517	−0.006	8	9	1.000000	No
		sgd	logistic	0.211	0.300	−0.089	16	32	0.029305	Degraded
			relu	0.211	0.300	−0.089	16	32	0.029305	Degraded
			tanh	0.211	0.300	−0.089	16	32	0.029305	Degraded
	KDEF	adam	logistic	0.714	0.727	−0.013	70	103	0.014738	Degraded
			relu	0.714	0.727	−0.013	70	103	0.014738	Degraded
			tanh	0.714	0.727	−0.013	70	103	0.014738	Degraded
		sgd	logistic	0.515	0.458	0.057	225	80	0.000000	Improved
			relu	0.515	0.458	0.057	225	80	0.000000	Improved
			tanh	0.515	0.458	0.057	225	80	0.000000	Improved

**Table 17:** Average Inference Throughput (FPS) Comparison over the Last 60 Detections for FFM-L03 and MobileNet Variants.

	FMM-L03	MobileNet Mini	MobileNet Big
Average FPS Last 60 detection	9.59	11.72	8.17

## Data Availability

Publicly available datasets were analyzed in this paper. These data can be found at: ISR-Coimbra. EmotionNet-6: Realistic Facial Emotion Dataset. Kaggle, 2025. Available online: https://www.kaggle.com/datasets/isrcoimbra/emotionnet-6-realistic-facial-emotion-dataset (accessed on 17 April 2026).

## Abbreviations

The following abbreviations are used in this manuscript:

FER	Facial Expression Recognition
FFM	Facial Feature Mapping
FM	Face Mesh
ML	Machine Learning
ICT	Information and Communication Technologies
QoL	Quality of Life
SVM	Support Vector Machine
DT	Decision Tree
RF	Random Forest
MLP	Multi-Layer Perceptron
PCA	Principal Component Analysis
t-SNE	t-Distributed Stochastic Neighbor Embedding
UMAP	Uniform Manifold Approximation and Projection
LASSO	Least Absolute Shrinkage and Selection Operator
HRI	Human–Robot Interaction
GDPR	General Data Protection Regulation
CNN	Convolutional Neural Network
RNN	Recurrent Neural Network
